# Differential expression pattern of an acidic 9/13-lipoxygenase in flower opening and senescence and in leaf response to phloem feeders in the tea plant

**DOI:** 10.1186/1471-2229-10-228

**Published:** 2010-10-25

**Authors:** Shouan Liu, Baoyu Han

**Affiliations:** 1 Tea Research Institute of Chinese Academy of Agricultural Sciences, Hangzhou, 310008, China; 2 China Jiliang University, Hangzhou, 310018, China

## Abstract

**Background:**

Lipoxygenase (LOXs) is a large family of plant enzymes that catalyse the hydroperoxidation of free polyunsaturated fatty acids into diverse biologically active compounds, collectively named phyto-oxylipins. Although multiple isoforms of LOXs have been identified in a wide range of annual herbaceous plants, the genes encoding these enzymes in perennial woody plants have not received as much attention. In *Camellia sinensis *(L.) O. Kuntze, no LOX gene of any type has been isolated, and its possible role in tea plant development, senescence, and defence reaction remains unknown. The present study describes the isolation, characterization, and expression of the first tea plant LOX isoform, namely *CsLOX1*, and seeks to clarify the pattern of its expression in the plant's defence response as well as in flower opening and senescence.

**Results:**

Based on amino acid sequence similarity to plant LOXs, a LOX was identified in tea plant and named *CsLOX1*, which encodes a polypeptide comprising 861 amino acids and has a molecular mass of 97.8 kDa. Heterologous expression in yeast analysis showed that CsLOX1 protein conferred a dual positional specificity since it released both C-9 and C-13 oxidized products in equal proportion and hence was named 9/13-CsLOX1. The purified recombinant CsLOX1 protein exhibited optimum catalytic activity at pH 3.6 and 25°C. Real-time quantitative PCR analysis showed that *CsLOX1 *transcripts were detected predominantly in flowers, up-regulated during petal senescence, and down-regulated during flower bud opening. In leaves, the gene was up-regulated following injury or when treated with methyl jasmonate (MeJA), but salicylic acid (SA) did not induce such response. The gene was also rapidly and highly induced following feeding by the tea green leafhopper *Empoasca vitis*, whereas feeding by the tea aphid *Toxoptera aurantii *resulted in a pattern of alternating induction and suppression.

**Conclusions:**

Analysis of the isolation and expression of the *LOX *gene in tea plant indicates that the acidic CsLOX1 together with its primary and end products plays an important role in regulating cell death related to flower senescence and the JA-related defensive reaction of the plant to phloem-feeders.

## Background

Phyto-oxylipins, which are among the important active components of plant cells, play diverse roles in several physiological events including plant growth and development, senescence, and defence against pathogens and pests as well as abiotic sources of stress [1,2]. Most phyto-oxylipins are derived from the lipoxygenase (LOX) pathway, which contains at least seven multi-enzyme branches. Of these enzymes, lipoxygenase is the first step and a key enzyme. Plant LOX (linoleate: oxygen oxidoreductase, EC 1.13.11.12) is a large family of non-heme iron-containing enzymes that catalyse the addition of molecular oxygen to linoleic acid (C18:2) and linolenic acid (C18:3) at either C9 or C13 position, with 9*S*- or 13*S*-hydroperoxides as the primary product, and hence are referred to as 9- or 13-LOX [1-3]. Plant LOXs can be further divided into two gene subfamilies, *type 1 *and *type 2*, based on the N-terminal chloroplast transit peptide sequence. The *type 1 *subfamily lacks a plasmid transit peptide and consists of both 9- and 13-LOXs, and the *type 2 *subfamily possesses 13-LOXs, which have a chloroplast transit peptide [1]. Both 9*S*- and 13*S*-hydroperoxides are rapidly converted into a series of biologically active molecules by the action of the LOX pathway enzymes, and are therefore referred to as phyto-oxylipins [1,2].

The highest LOX activity was observed in soybean during leaf growth [4], and LOX activity level was reported as being positively correlated with the rate of elongation of an organ [5]. Potato *LOX1 *is expressed during tuber growth, and its antisense suppression resulted in smaller tubers [6]. Maize LOX3 (*ZMLOX3*) knockout mutants showed shorter roots and increased senescence [7]. The olive 9/13-LOX gene is predominantly expressed during fruit ripening and is believed to be associated with senescence in the plant [8]. A similar observation was made in kiwifruit LOXs [9]. Some LOXs, such as *r9-LOX1 *and *OsLOX1 *in rice [10,11] and *PdLOX *in almond [12], were found to be involved in germination. However, there is little information on the role of LOXs in flower bud opening and petal expansion. Physiological and biochemical analyses show that lipid content changes and the cellular membrane is degraded during flower senescence [13,14] and that lipid oxidation is either due to increased LOX activity or independent of LOX [15]. Increased LOX activity prior to obvious senescence of flowers has been shown in many plants including carnation [16], day lily [17], and rose [18]. In *Petunia inflata*, a highly up-regulated allene oxide synthase (AOS) gene involved in petal senescence was identified and assumed to have a role in programmed cell death [19] whereas in *Alstroemeria peruviana *and two orchid species, no increase in LOX-specific activity was observed over time [20,21]. It is likely that the function of LOXs during petal senescence varies from species to species [22] and needs further study.

The expression LOX genes is most frequently observed in the response of plants to pest attack, particularly that of many annual herbaceous plants to insects [1,23]. Transcripts encoding *LOX *genes were strongly induced during plant-aphid interactions including *Myzus euphorbiase *feeding on tomato leaves [24] and *M*. *nicotianae *sucking the sap from *Nicotiana attenuata *[25]. The cabbage *BoLOX *was up-regulated by several herbivores such as caterpillars (*Pieris rapae*, *P. brassicae*, and *Mamestra brassicae*), spider mites (*Tetranychus urticae*), and locusts (*Schistocerca gregaria*) but not by aphids (*M. persicae*) [26]. In rice, two LOX genes are involved in the plant's response to stress brought about by herbivores: the *OsLOX1 *transcript was rapidly and abundantly induced by the brown planthopper (BPH) *Niaparvata lugens *[11], and the *OsHI-LOX*, which was expressed only slightly by BPH but strongly by a chewing insect, namely the striped stem borer (SSB) *Chilo suppressalis *[27]. The antisense-mediated depletion of LOX gene suggests that its role in plant defence varies with the type of herbivore. Silencing the *NaLOX3 *made *N. attenuata *unable to produce defensive compounds and hence more susceptible to the chewing insect *Manduca sexta *[28]; however, in the field the transformed plants are susceptible to many herbivores, including a new opportunistic phloem-feeder, the leafhopper *Empoasca *sp., which does not select the wild-type *N. attenuata *[29]. Transgenic rice with sense or antisense *OsLOX1 *would increase or decrease the resistance to BPH [11]. It is noteworthy that antisense expression of rice *OsHI-LOX *encourages chewing herbivores but deters the phloem-feeder [27]. LOX genes are less commonly observed in woody perennials than in herbaceous annuals. The tea plant, being a perennial, is exposed to pests for longer time and being larger, presents a greater expanse for pests -- a study of expression patterns of its LOX genes in response to attack by herbivores therefore assumes importance.

The tea plant, *Camellia sinensis *(L.) O. Kuntze, is not only one of the world's most important woody plantation crops but is also valued as a source of secondary metabolic products including phyto-oxylipins. Tender tea buds and leaves are usually plucked and processed to produce high-grade tea as a beverage. Tea flowers emit plenty of aromatic volatiles to attract bees and wasps and have become a major source of honey [30]. However, tea is also attacked by herbivores such as tea green leafhoppers and aphids. As a defence mechanism, tea can emit specific volatile organic compounds (VOCs) or change the relative proportions of many components of the VOCs, which may attract predators and parasites - natural enemies of the herbivores - to keep the pests in check [31,32]. As the first step to elucidating the function of tea plant oxylipins in development and defence, we worked on isolating and characterizing a tea plant LOX gene, namely *CsLOX1*. Purification and biochemical analysis of the recombinant protein showed that CsLOX1 is a dual position-specific lipoxygenase producing both C-9 and C-13 HPOs. The *CsLOX1 *was expressed predominantly in flowers, but differentially regulated during flower opening and senescence. We also studied the dynamics of *CsLOX1 *expression patterns in leaves following mechanical wounding, hormone treatment, and attack by two phloem-feeders (tea green leafhopper and tea aphid) and the role of *CsLOX1 *in flower development and defence against insect attack.

## Results

### Isolation and sequence analysis of CsLOX1

To clone the *C. sinensis *lipoxygenase gene, three PCR fragments were first amplified by RT-PCR reaction using degenerate primers that correspond to the conserved amino acid sequences from other plant LOXs. The resulting segments were 298 bp, 328 bp, and 544 bp long, and their sequences showed a high degree of identity to lipoxygenase from other plants. The cDNA ends were then amplified with 5' and 3' RACE-PCR. Finally, the cDNA sequences were combined and validated by PCR amplification with the primers derived from 5' and 3' RACE products. As this is the first lipoxygenase gene reported in the tea plant, the gene was named *CsLOX1 *(Genbank: EU195885). The gene was approximately 2796 bp long, comprising a 191 bp long 5'-untranslated region (UTR) and a 73 bp long 3'-UTR with poly (A) signals, and contained an open reading frame (ORF) of 2586 bp encoding a presumed translation product of 861 amino acids with an M_r _of 97.8 kDa and a pI of 5.74. Based on the BLAST P network service, the predicted amino acid sequence of *CsLOX1 *showed a high degree of identity with other plant LOXs such as common olive (77.9%, Genbank: EU678670), almond (77.2%, Genbank: AJ404331), potato (77%, Genbank: U60202), and hazelnut (76.9%, Genbank: AJ417975), which belong to *type 1*, but the gene also shared its identity to a small extent with LOX isoforms from the *type 2 *group. Sequence analysis of *CsLOX1 *showed several functional domains typical of plant LOXs: (a) the conserved domains involved in substrate binding ("AWRTDEEFAREMLAG", positions 361-375) and oxygen binding ("ASALHAAVNFGQY", 709-721), (b) the highly conserved C-terminal amino acid sequence ("GIPNSVSI", 854-861), (c) the plant LOXs position specificity motif for 9-LOX ("R/TV" at positions 730/579-580) (which was present at the active site, but SP-HPLC analysis of recombinant *CsLOX1 *products later indicated it to be a non-conventional LOX with 9/13-LOX activity), (d) five highly conserved histidine residues, one each at positions 517, 522, 527, 545, and 554, and (e) amino acid residues essential for the ability to bind iron (His 517, His 527, His 713, Asn 717, and Ile 861) at the active site (Additional file 1).

**Figure 1:**
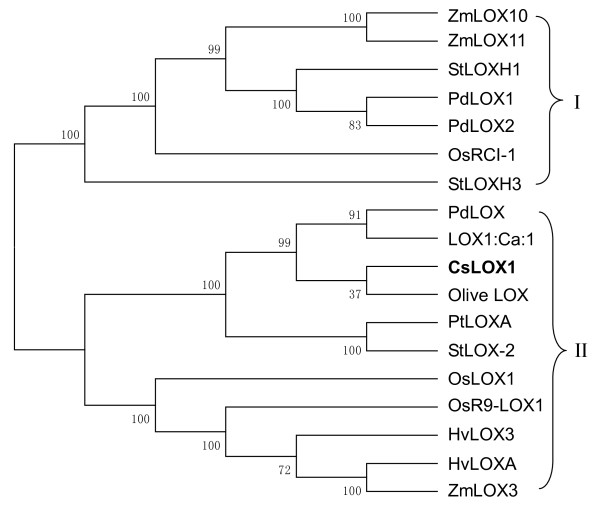
**Phylogenetic analysis of *CsLOX1 *and selected plant LOXs showing clustering within two groups**. Plant LOXs in group I were localized in the chloroplast with a chloroplast transit peptides and group II LOXs were localized in the cytoplasm. The alignment of amino acid sequences of the LOXs was performed using ClustalW. The phylogenetic tree was constructed by neighbour-joining method using MEGA software. Accession numbers of the sequences used to build the tree were as follows: maize: *ZmLOX3 *(AF329371), *ZmLOX10 *(DQ335768), and *ZmLOX11*(DQ335769); potato: *StLOXH1 *(T07062), *StLOXH3 *(T07062), *PtLOXA *(X95513), and *StLOX-2 *(Y18548); poplar: *PdLOX1 *(DQ131178) and *PdLOX2 *(DQ131179); rice: *OsRCI-1 *(AJ270938), *OsLOX1*(DQ389164), and *OsR9-LOX1*(AB099850); almond: *PdLOX *(AJ404331); hazelnut: *LOX1:Ca:1*(AJ417975); tea plant: *CsLOX1 *(EU195885); olive: *LOX *(EU678670); and barley: *HvLOX3 *(L37358) and *HvLOXA *(L35931).

The phylogenetic relationships among the deduced amino acid sequences of *CsLOX1 *and those of other reported plant LOXs were determined by constructing a phylogenetic tree (Figure 1). Neighbour-joining analysis identified two groups of plant LOX proteins that appeared to reflect accurately their subcellular localization. LOXs were classified as follows: group I was found in the chloroplast with chloroplast transit peptides at the N-terminal and belonged to *type 2 *subfamily, and group II was found in the cytoplasm and belonged to *type 1 *subfamily. *CsLOX1 *belongs to group II, *type 1 *gene family, and is predicted to be in the cytoplasm. Other LOXs from group II, such as *ZmLOX3 *[7,33], *OsLOX1 *[11], and *PdLOX *[12], are reported as being involved in the defence mechanism, whereas olive *LOX *is reported to be involved in fruit ripening and senescence [8].

### Biochemical activity and characterization of recombinant yeast CsLOX1

**Figure 2:**
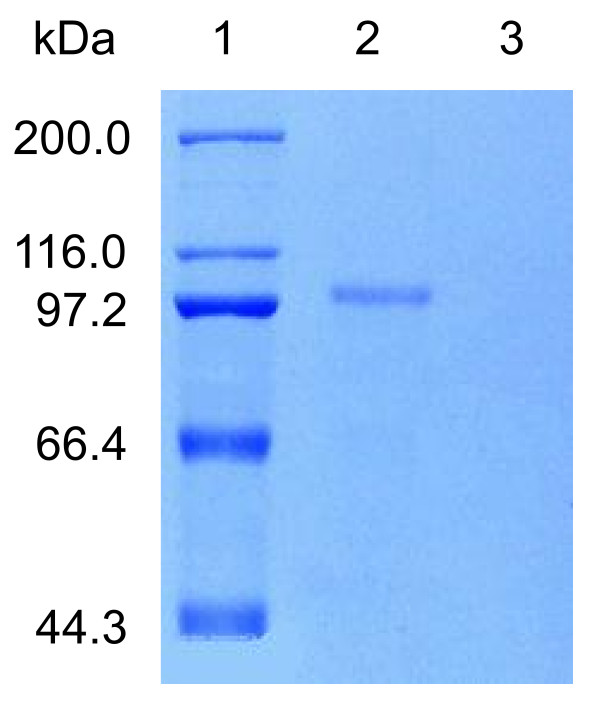
**SDS-PAGE analysis of the recombinant CsLOX1**. The proteins were stained with Coomassie brilliant blue R-250. 1, Positions of marker proteins. 2, SDS-PAGE of the purified CsLOX1 extracted from pPIC9K/*lox1 *transformed yeast cultures (10 μg). 3, SDS-PAGE of the extracts from empty pPIC9K transformed yeast cultures.

The ORF of *CsLOX1 *was cloned to multiple cloning sites of the expression vector pPIC9K, and a recombinant plasmid, namely pPIC9K/*lox1*, was constructed. The linearized plasmid pPIC9K/*lox1 *was then transformed to *Pichia pastoris *GS115 and over-expressed. The CsLOX1 protein with biochemical activity was synthesized and secreted to the culture medium when a recombinant yeast strain was induced by 0.5% methanol. The recombinant lipoxygenase encoded by *CsLOX1 *was purified and characterized. The molecular mass of recombinant CsLOX1 was represented by a unique band of expected size (approximately 98 kDa) in Coomassie brilliant blue stained SDS-PAGE whereas no such similar band was seen in induced control strains harbouring the empty vector (Figure 2).

**Figure 3:**
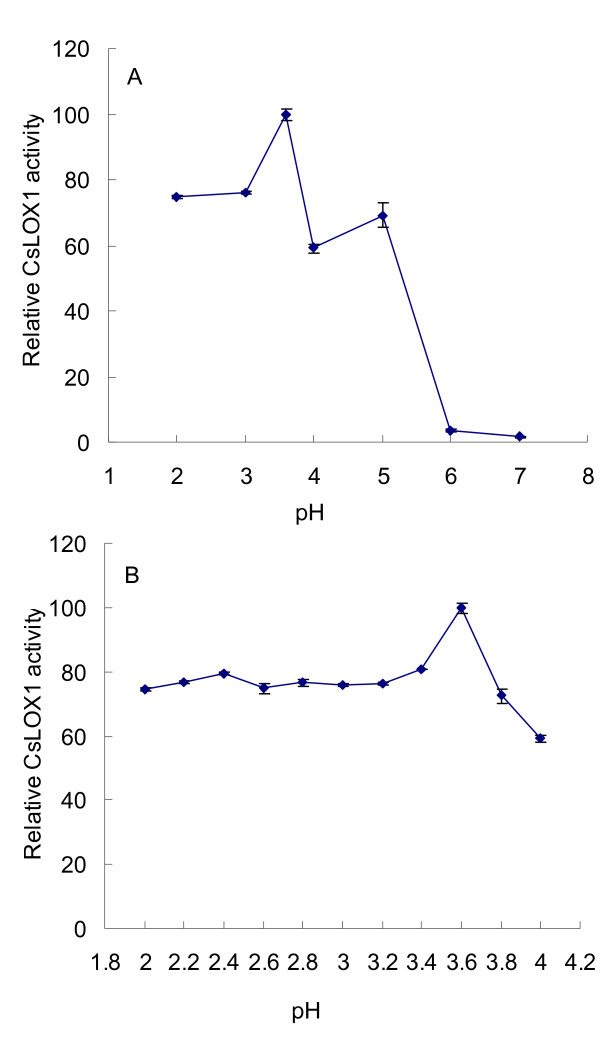
**The optimum pH for recombinant CsLOX1**. A and B: The optimum pH for the recombinant CsLOX1. The maximum activity was estimated as 100%, and all the experiments were replicated three times. Means ± SE were obtained from three independent experiments.

Biochemical activity of the purified recombinant CsLOX1 was investigated by measuring the increase in *A*_234 _using linoleic, linolenic, and arachidonic acids as substrates. The optimum pH was determined by varying the pH values of the reaction buffers from 2.0 to 8.0. At 25°C, the optimum pH turned out to be 3.6 (Figure 3A) since CsLOX1 showed the highest activity (16.2 units/mg protein with linoleic acid as substrate) at that pH. The enzyme activity was also fairly high (about 70%) at the pH range of 2.0 to 3.8 (Figure 3B). However, the activity dropped to 7.78 units/mg protein and 2.16 units/mg protein with linolenic and arachidonic acids as substrates, respectively.

The kinetic parameters were analysed using linoleic and linolenic acids as substrates (Table 1). The recombinant CsLOX1 showed 4.29-fold higher *Km *value for linoleic acid (141.12 μM) than for linolenic acid (32.89 μM). Comparison of the *Vmax *values showed that CsLOX1 oxidized linoleic acid approximately 6 times faster than linolenic acid did. The *kcat/Km *values of recombinant CsLOX1 for linoleic acid and linolenic acid hydroperoxidation were 38.55 and 26.72 s^-1 ^μM^-1^, respectively. These results indicate that linoleic acid is clearly the preferred substrate for the recombinant CsLOX1.

**Table 1:** Kinetic parameters of purified recombinant CsLOX1.

Substrate	Km (μM)	**Vmax (μmol s**^**-1**^**)**	**kcat (s**^**-1**^**)**	**kcat/Km (s**^**-1**^**μM**^**-1**^**)**
Linoleic acid	141.12	41.16	5440.02	38.55
Linolenic acid	32.89	6.65	878.91	26.72

**Figure 4:**
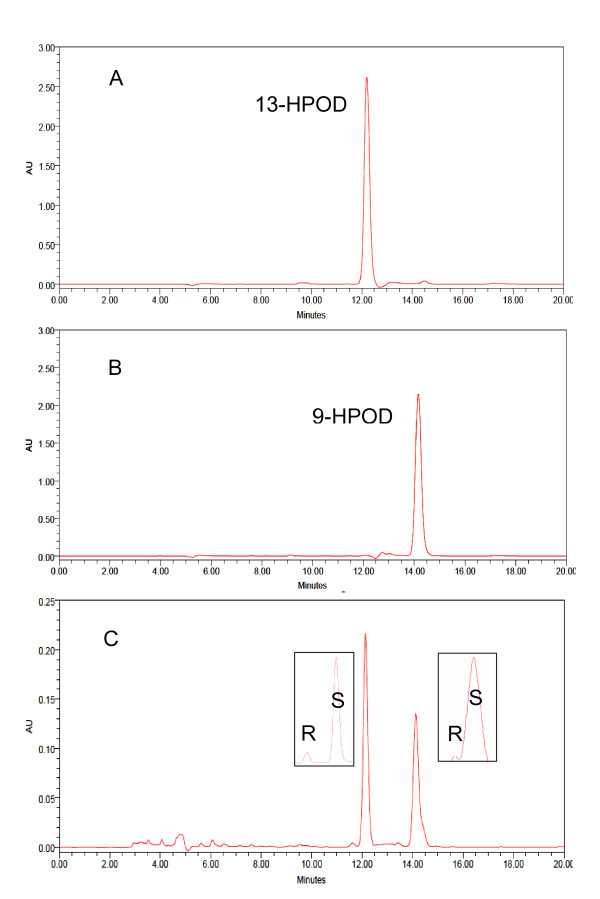
**Determination of positional specificity of the recombinant CsLOX1**. A and B show the retention time of the isomers produced by soybean LOX1 compared with authentic standards of 13-HPOD and 9-HPOD. C, SP-HPLC analysis of the reaction mixture catalysed by recombinant CsLOX1. Boxes: chiral-phase HPLC showing the enantiomer composition of 9- and 13-HPOD.

In order to study the position-specificity of recombinant CsLOX1, we separated the reaction products by SP-HPLC. The retention times of CsLOX1 products were consistent with authentic standards of both 9- and 13-HPOD and those of soybean LOX1 (Figure 4A, B). CsLOX1 produced both 9-HPOD and 13-HPOD in equal proportion in the reaction mixture (Figure 4C). This observation is inconsistent with the positional specificity rule of plant LOXs, namely that R/TV motif at the active site indicated the 9-LOX [1]. Although R/TV motif was found at positions 730, 579, and 580, the CsLOX1 products contained 9-HPOD as well as 13-HPOD. In this study, it was evident that *CsLOX1 *was a dual-position-specific lipoxygenase and was accordingly named tea plant 9/13-CsLOX1. The stereochemistry of the reaction products was analysed by chiral-phase HPLC. 9-HPOD and 13-HPOD were predominantly in the *S *configuration, indicating that they had been derived from the activity of a specific enzyme (Figure 4C).

### Expression of CsLOX1 in different tissues

**Figure 5:**
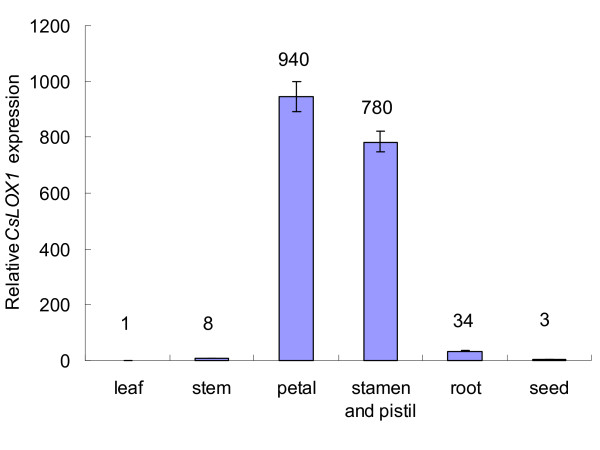
**Expression analysis of *CsLOX1 *in different tissues**. Relative expression levels of *CsLOX1 *in tea plant tissues. Total RNA from tea leaves, stems, petals, stamens and pistils, roots, and seeds was used for real-time RT-PCR. The *CsLOX1 *expression level in leaf was 1.

To study the tissue specificity of *CsLOX1*, quantitative RT-PCR was used for examining its expression pattern in the root, stem, leaf, flower (petal, stamen, and pistil), and seed. Transcript abundance of *CsLOX1 *was the highest in flower and the lowest in the rest (Figure 5). Petals showed the highest transcript levels, about 940-fold higher than those in leaves. A similar expression pattern in floral tissues has been reported in pea *LOX *[34], peanut *PnLOX1 *[35], and rose *RLOX1 *[18]. In rose, the transcript of the *RLOX1 *gene was dramatically increased in response to senescence of petals, and the gene was held to play a role in promoting petal senescence [18].

### Total LOX activity during flower bud opening and senescence

**Figure 6:**
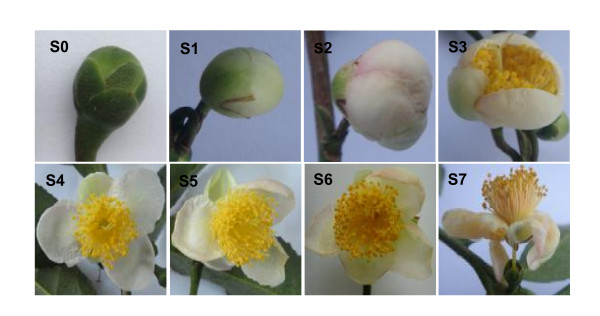
**Tea flower bud development, opening, and senescence**. S0, bud maturing but yet green; S1-S4 for flower bud opening stages; S5-S7 for flower senescence stages.

Since earlier reports suggest that LOXs are involved in regulating plant growth and development [23], we analysed total LOX activity *in vitro *during flower bud opening and senescence in eight stages, which were designated as follows (Figure 6): S0, buds maturing but yet green; S1, buds slightly white, closed, and with firm tips; S2, buds white, expanded, with soft tips but not yet open; S3, half-opened flowers; S4, just opened flowers, fully open with petals perfectly horizontal; S5, fully open flowers 24 h after S4; S6, fully open flowers about 48 h after S4 with petals showing initial signs of senescence; S7, fully open flowers about 72 h after S4 with clearly rolled-in petals and separated from the plant together with stamens.

**Figure 7:**
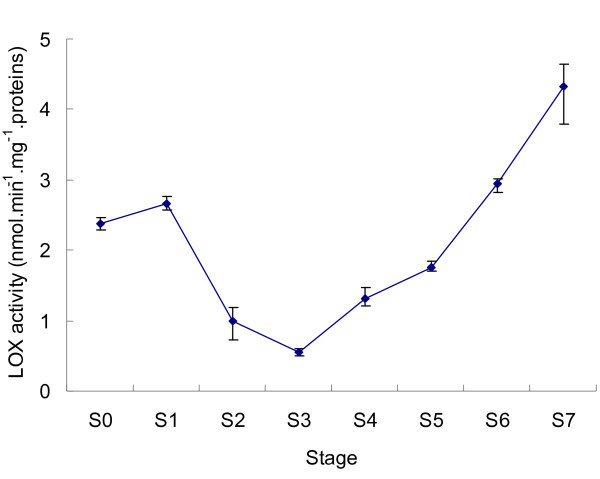
**Changes in activity of lipoxygenase during different stages of tea flower development**. Total proteins extracted from tea flowers at different stages were used for analysing LOX activity. Values represent the means and SE.

The total LOX activity during opening and senescence had at least two clearly different phases, the decreasing phase and the increasing phases (Figure 7). In the first phase, when the buds were opening, total LOX activity declined by about 80% from S1 to S3; in the second phase, the activity increased slowly from S3 to S5 and then more rapidly to S7 and was about 8-fold higher than that in S3. During this process, the flower opened fully and then senesced gradually; total LOX activity began to increase even before the petals had fully expanded. LOX activity also increased slightly from S0 to S1, when the bud was developing. Taken together, the LOX activity was relatively high when floral buds were tightly closed, which may indicate that they were in the process of development, declined to the lowest level during flower opening, and began to increase again, peaking at senescence. In *A. peruviana *too, total LOX activity declined when the buds were opening [20] but differed from the present case in that it continued to decline right up to senescence when the perianth abscised. In this study, LOX activity during flower senescence was similar to that in day lily petals in that the activity began to increase even before flower opening and then increased more rapidly during petal senescence [17].

### Expression analysis of CsLOX1 in flower bud opening and senescence

**Figure 8:**
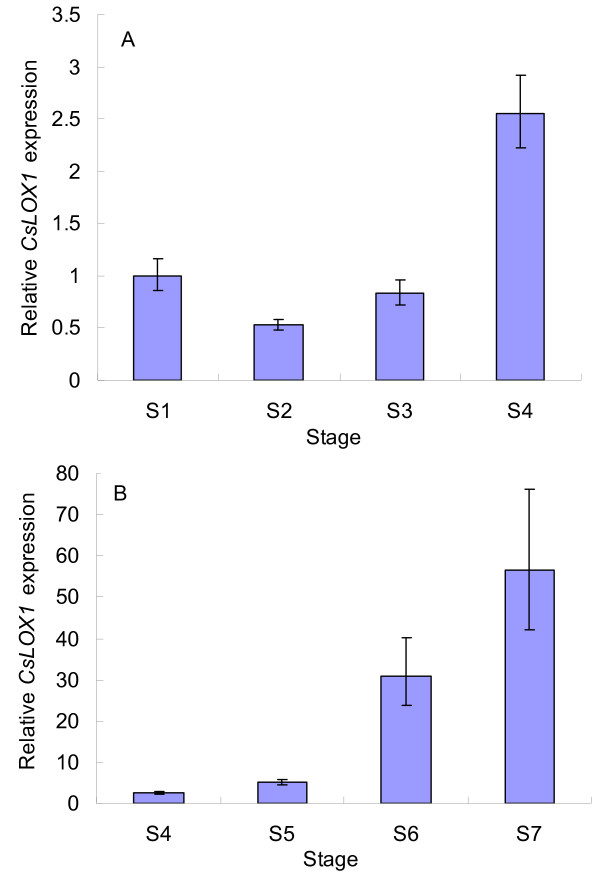
**Expression of the *CsLOX1 *transcripts during flower opening and senescence**. Real-time RT-PCR analysis of the *CsLOX1 *relative expression levels in tea petals at different stages. (A) Total RNA from S1 to S4 was used for real-time RT-PCR. (B) Total RNA from S4 to S7 was used for real-time RT-PCR. The *CsLOX1 *relative expression level in S1 was set to 1. Values represent the means and SE.

To find out whether the *CsLOX1 *gene is involved in tea flower opening and senescence, real-time quantitative RT-PCR was carried out. Transcript analysis of *CsLOX1 *during flower bud opening showed that gene expression decreased after S1 and remained low until S4, during which the petals opened fully (Figure 8A).

Transcripts of the *CsLOX1 *gene increased dramatically in tea petals from S5 to S6 and from S6 to S7, when the petals reached senescence and withered (Figure 8B). In other words, the gene was down-regulated during petal opening and up-regulated after the petals had opened fully, especially during petal senescence. The pattern of expression of the *CsLOX1 *gene during flower bud opening and senescence was similar to that of total LOX activity observed during the same stages. The *CsLOX1 *gene expression level during S1 was only 1.76% of that during S7 but the total LOX activity during S1 was approximately 61.5% of that during S7 and therefore of comparable magnitude. As the tea plant had at least four *LOX *genes (data not shown), it is likely that total LOX activity during S1 involved other lipoxygenase isoforms as well in flower development, whereas the *CsLOX1 *gene is expressed during development as well as during senescence and plays a preeminent role in senescence.

### Effect of MeJA, SA, and mechanical damage on CsLOX1 transcription

**Figure 9:**
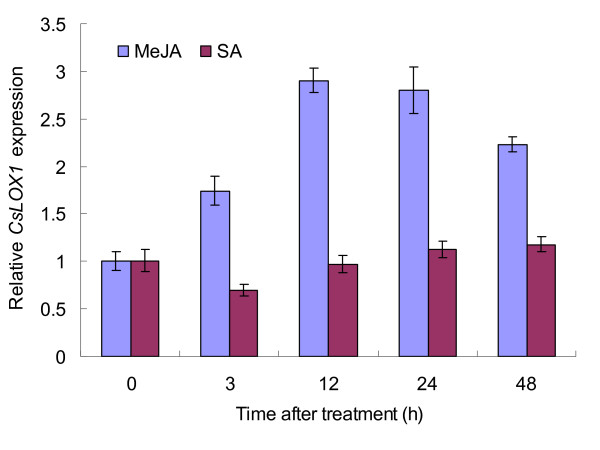
**Response of the *CsLOX1 *gene to MeJA and SA treatments**. Real-time RT-PCR analysis of the relative expression of *CsLOX1 *in tea leaves at different times after MeJA (200 μM) and SA (2.5 mM) treatments. Expression level in untreated samples was taken as 1; values represent the means and SE.

**Figure 10:**
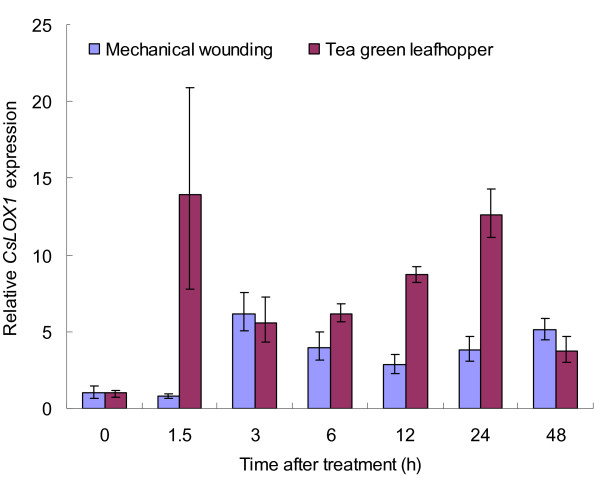
**Response of *CsLOX1 *gene to mechanical wounding and feeding by the tea green leafhopper**. Real-time RT-PCR analysis of the relative expression of *CsLOX1 *in tea leaves at different times after mechanical wounding and after feeding by the tea green leafhopper. Expression level in untreated samples was taken as 1; values represent the means and SE.

In leaves, exogenous MeJA induced the expression of the *CsLOX1 *gene (Figure 9). Maximum expression of *CsLOX1 *after the MeJA treatment was at 12 h and decreased thereafter, although it remained higher than that in the control at 48 h. Unlike MeJA, exogenous SA did not increase *CsLOX1 *mRNA accumulation but suppressed it slightly (Figure 9). Transcripts of *CsLOX1 *accumulated rapidly to reach a high level 3 h after mechanical damage but decreased at 6 h and 12 h following the injury--only to increase again at 24 h to reach an even higher level at 48 h (Figure 10). Thus *CsLOX1 *appears to be up-regulated by wounding and MeJA but not by SA.

### Expression analysis of CsLOX1 in response to phloem feeders

To explore the role of *CsLOX1 *in mitigating the stress occasioned by herbivores, two different phloem feeders, namely the tea green leafhopper and the tea aphid, were placed on tea leaves.

In leaves eaten by leafhoppers, *CsLOX1 *transcripts increased rapidly to the highest level (approximately 14-fold more than that in the control) 1.5 h after exposure to the pest but declined to about 60% of that in the highest level at 3 h; after 3 h, the level increased again until 24 h and declined thereafter to 70% of the highest level at 48 h, but still remained higher than control (Figure 10).

**Figure 11:**
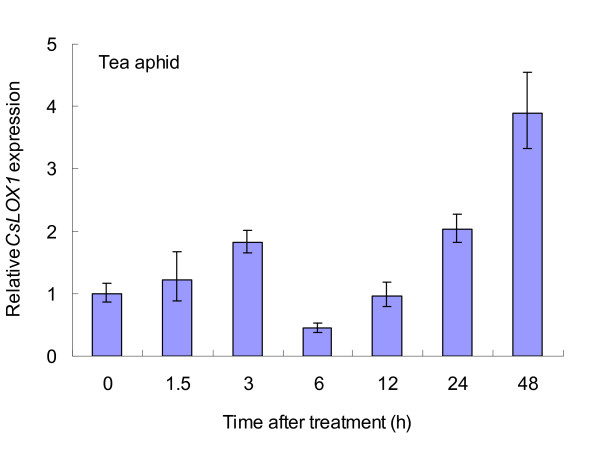
**Response of *CsLOX1 *gene to feeding by the tea aphid**. Real-time RT-PCR analysis of the relative expression of *CsLOX1 *in tea leaves at different times after attack by the tea aphid. Expression level in untreated samples was taken as 1; values represent the means and SE.

In the case of the tea aphid, *CsLOX1 *transcript pattern fell into three phases (Figure 11): in the first phase, the expression increased and the gene was up-regulated within the first 3 h, similar to the results of mechanical wounding and the MeJA treatment; in the second phase, the expression declined sharply and the gene was down-regulated at 6 h and 12 h, suggesting that the aphids had suppressed gene expression at that phase; in the third phase, 24 h and 48 h after the treatment, the gene was up-regulated again and the expression increased to its highest level. Triggering of the transcription and a rapid increase in its expression level were the most frequently observed responses to insect attack [3], a pattern consistent with that observed by earlier workers, namely that some *LOXs*, together with other defence-related genes, decrease their expression during infection [36].

### Analysis of LOX-derived volatiles from intact leaves and from those damaged by different insects

**Figure 12:**
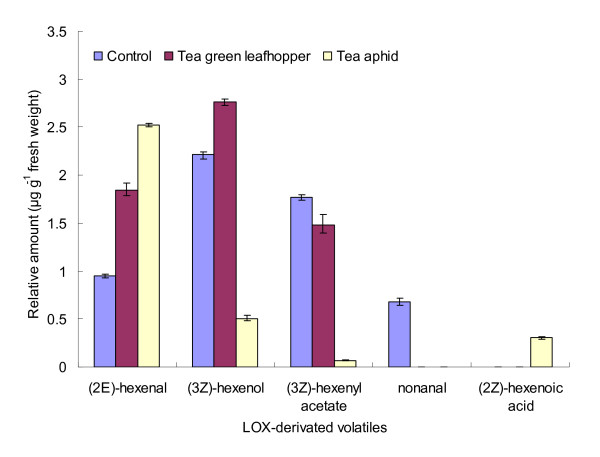
**Relative amount of LOX-derived volatiles emitted by the control plants and by plants infested with the tea green leafhopper and the tea aphid**.

To ascertain the possible involvement of *CsLOX1 *in the synthesis and emission of green-leaf volatiles in the tea plant as a defensive response to attack by insects, the profiles of LOX-derived volatile compounds obtained from intact plants and from those damaged by the tea green leafhopper and the tea aphid were analysed and compared. The LOX-derived volatiles consisted of C-6 and C-9 aldehydes, alcohols, and esters. As shown in Figure 12, intact tea plants released nonanal, a C-9 aldehyde, but it was not detected in plants attacked by the phloem feeders. On the other hand, the C-6 volatiles, namely (3Z)-hexenol, (2E)-hexenal, and (3Z)-hexenyl acetate, were the main components of the volatiles released by the infested plants.

In the case of the leafhoppers, the infested plants released greater quantities of (3Z)-hexenol and (2E)-hexenal and slightly lower quantities of (3Z)-hexenyl acetate than the intact plants did. This pattern correlated with the increase in the expression level of *CsLOX1 *in plants attacked by the leafhoppers (Figures 10 and 12).

In the case of the tea aphids, the infested plants released lower quantities of (3Z)-hexenol and (3Z)-hexenyl acetate but greater quantities of (2E)-hexenal and also released (2Z)-hexenoic acid. These results suggest that some LOX-derived volatiles were suppressed and some others were induced as a result of aphid infestation. The emission of LOX-derived volatiles by aphid-infested plants correlated with the expression pattern of *CsLOX1 *(Figures 11 and 12). In addition, the increased levels of (2E)-hexenal following aphid infestation were reported to be part of a tritrophic interaction wherein the volatile attracts the predators and parasites of the tea aphid such as *Leis axyridis*, *Chrysopa septempunctata*, and *Aphidius *sp., and thus plays an indirect role in the plant's defence mechanism [30,31].

## Discussion

The similarities in amino acid sequences of other plant LOXs and those of *CsLOX1 *were examined to identify the gene. The predicted gene included all the typical plant LOX domains but lacked an N-terminal transit peptide. Phylogenetic analysis showed that *CsLOX1 *can be characterized as a *type 1 *LOX gene, which is commonly found in the cytoplasm. The gene appears to be closely related to the LOXs in olive, almond, potato, and hazelnut, but shares few similarities with *type 2 *LOXs, which are known to be located in chloroplasts. After the *CsLOX1 *gene was introduced into yeast *P*. *pastoris*, it was heterologously expressed and its products showed lipoxygenase activity but showed characteristics that differed from those of other plant LOXs. Generally, plant LOXs position-specificity is determined by the primary motifs R/TH, R/TF, R/SF, or R/CF for 13-LOX and R/TV for 9-LOX at the active site [1]. The *CsLOX1 *contained an R/TV motif and was predicted to be 9-LOX. Surprisingly, as shown by HPLC analysis, both 9- and 13- hydroperoxides of linoleic acid, predominantly in the *S *configuration, were produced in equal proportion by the recombinant CsLOX1, a feature that did not fit the predictive position-specificity models; similar results were observed in some other plant LOXs such as potato *StLOX-2 *[37], maize *ZMLOX1 *[38], hazelnut *LOX1:Ca:1 *[39], pea *LOXN2 *[40], rice *OsLOX1 *[11], and olive *LOX *[8]. These enzymes displayed a dual-positional-specificity and may be named 9/13-LOXs. Within the 9/13-LOXs group, maize *ZmLOX1 *responded to wounding and MeJA, but MeJA induced *ZmLOX1 *expression exhibiting a biphasic pattern comprising an early phase and a late phase [38]; rice *OsLOX1 *was induced by wounding and was involved in defence against BPH [11]; and olive *LOX *was mainly expressed during late developmental stages, and may be associated with fruit ripening and senescence [8]. It was reported that LOX specificity has implications for hydroperoxide metabolism, and 9/13-LOX products such as 9-and 13-hydroperoxide from linoleic and linolenic acids can be converted into volatile aldehydes by hydroperoxide lyase (HPL), whereas 13-hydroperoxide from linolenic acid can be converted into JA cyclic precursors by AOS [1,2,8]. These results suggest that 9/13-CsLOX1 is involved in diverse biological branches of the LOX pathway and is able to produce compounds that play important roles in a plant's development and in its defence response [5].

The optimal pH for recombinant CsLOX1 activity was 3.6, which might be the lowest reported for any plant LOX to date, including pea *LOXN2 *(4.5) [40], rice *OsLOX1 *(4.8) [11], rose *Rlox1 *(4.8) [18], and carnation soluble *LOX *isoforms (4.9-5.8) [41]. The acidic LOXs from carnation and rose petals are believed to play an important role in flower senescence by way of membrane disruption by the HPOs and free radical action [18,41]. However, in processes other than senescence, especially during flower opening, these potentially harmful components and other reactive oxygen species (ROS) should be prevented from acting. Total LOX activity decreased during the flower opening stage in *A. peruviana *[20], and endogenous H_2_O_2 _decreased and the specific activity of protective enzymes increased or remained at a higher level than that at senescence in day lily [17]. Our results showed that *CsLOX1*, together with the flower LOX activity, was suppressed during flower bud opening and high levels of it were induced during petal senescence. These results indicate that *CsLOX1 *is involved in flower development, playing a regulatory role in flower senescence.

Analysis of tissue-specific mRNA accumulation showed that *CsLOX1 *levels were the lowest in leaves. *CsLOX1 *was induced and its transcripts accumulated above the basal level following mechanical wounding or MeJA treatment, but SA treatment did not induce *CsLOX1*. Two LOX genes in poplars (*PdLOX1 *and *PdLOX2*) were also up-regulated by mechanical wounding and exposure to MeJA, but both were down-regulated by SA. The genes were reported to be involved in defending poplars against the necrotrophic pathogen *Marssonina brumnea *f. sp. *Multigermtubi *[42]. Also, the rice *OsHI-LOX *gene expression levels were induced by mechanical damage and JA treatment but not by SA, and the gene was proved to have a role in herbivore-induced JA biosynthesis [28]. *CsLOX1*, together with the above genes, seems to be involved in JA-related plant defence and that against attacks by herbivores.

On the basis of LOX-derived volatiles, it was inferred that the LOX pathway is activated mainly following infestation by the tea green leafhopper whereas in aphid-infested plants, the LOX pathway is not only induced inadequately but also positively suppressed. Concerning expression of the *LOX *gene in response to attack by the tea green leafhopper and the tea aphid, it appeared that although the gene had different transcript patterns, each had a clear correlation with the emission pattern of LOX-derived volatiles. Tea green leafhopper is a highly mobile, opportunistic phloem-feeding herbivore, and the gene's response to sucking by the insect is transient and biphasic: The first phase begins within about 1.5 h after the insect begins feeding, and the highest transcript level is reached during this phase; the second phase begins 3 h after the feeding has started, and the expression level continues to be fairly high in this phase. In the case of mechanical wounding, the pattern of expression is similar although the transcript level is lower (Figure 10): *CsLOX1 *accumulates rapidly at 3 h and also has two phases. Pea *LOXN2 *exhibits similar expression patterns in response to wounding [40] and maize *LOX1*, to exogenous MeJA treatment [38]. These LOXs function in different phases of the response to stress and are deployed quickly in response to cell damage due to oxidative burst [38,40], decline rapidly thereafter, and either remain at the elevated level throughout the defensive response or return to their normal levels in tissue development. Oral secretions of the tea aphid are produced over a longer time than those of the leafhopper and the aphid also continues feeding for much longer [43,44]. Accordingly, the aphid elicits a multi-stage response, which comprises two up-regulation stages separated by a suppression stage (Figure 11). Some aphids strongly induce LOX transcripts and elicit JA-mediated resistance [45] but others do not [26,46]. On the other hand, some aphids use a decoy strategy to suppress an effective JA response by inducing SA-dependent defence in a variety of plant species [47,48]. These reports indicated that the role of JA-related LOXs in resistance to aphids varies from one plant species to the next. Our results showed that MeJA induction of *CsLOX1 *gene in the tea plant following aphid attack is more complicated, as revealed by the alternating stages of induction and suppression of the gene. The tea aphid may use its oral secretions while feeding on tea leaves to neutralize some effective defence mechanism involving JA whereas with the tea green leafhopper, the plant has to deal mostly with wounding, and JA-related resistance may prove adequate. These aspects, however, need further investigation.

## Conclusions

Based on similarities in the sequence of amino acids, we have identified a LOX pathway gene, *CsLOX1*, the expression level of which in petals was 940 times greater than that in leaves. The gene showed opposing regulation patterns during flower development: the expression was down-regulated during flower opening but strongly up-regulated during petal senescence. In leaves, the gene was up-regulated following wounding and MeJA treatments and was also induced in response to feeding by the tea green leafhopper but suppressed in response to feeding by the tea aphid at some stage of the protracted and complicated interaction between the plant and the aphid. When the *CsLOX1 *gene was over-expressed in yeast, the purified protein showed unusual catalysis over a broad acidic pH range of 2.0 to 3.8 and displayed dual positional specificity with 9/13-LOX activity. Phylogenetic analysis indicated that the 9/13-LOX plays diverse roles in plant development and the plant's defence mechanisms. Further investigation aimed at evaluating other LOXs or genes downstream of the LOX pathway will help in understanding the function of tea plant oxylipins in defence and development.

## Methods

### Plant materials

Mature seeds of tea (*Camellia sinensis ***'**Longjing 43') were harvested from the experimental field of the Tea Research Institute, Chinese Academy of Agricultural Science (TRI, CAAS), Hangzhou, China, and plants from the seeds were grown in a greenhouse under controlled conditions (50%-70% relative humidity, 20-30°C, and 12 h of light alternating with 12 h of darkness). Tea plants with six to eight leaves were used for the experiments. For herbivore infestation, the plants were placed in a climate chamber (70 ± 10% relative humidity, 25 ± 2°C, and the same photoperiod as mentioned above). Flowers harvested at different developmental stages were used for analysing LOX activity and petals from the flowers were used for analysing gene expression.

### MeJA, SA, and wounding treatments of leaves

MeJA (Sigma, 95%) and SA were diluted to their final concentrations of 200 μM and 2.5 mM, respectively, with sterile water containing 0.01% Tween-20. The solutions were sprayed onto tea leaves; leaves sprayed only with 0.01% Tween-20 served as a control. For the wounding treatment, leaves were pricked with a sterilized pin. All treated leaves were harvested at different times after the treatment, frozen quickly in liquid N_2_, and stored at -70°C until required.

### Infestation with the tea green leafhopper

Ten tea plants with tender leaves were deliberately infested with about 100 nymphs of the tea green leafhopper, and each plant was covered with a small mesh bag to prevent the nymphs from moving to other tea plants. Infested leaves were collected at 1.5, 3, 6, 12, 24 and 48 h after the beginning of the infestation. Healthy tea leaves free of any infestation were harvested as a control. The harvested leaves were frozen in liquid N_2 _and stored at -70°C. Volatiles were collected from plants that had been infested by the tea green leafhopper for 24 h.

### Infestation with the tea aphid

The method was the same as that described above in every respect except that 500 aphids were used for infestation. The aphids were removed with a soft brush before harvesting.

### Isolation of full-length CsLOX1 cDNA

Leaves that had been exposed to biotic or abiotic sources of stress were used for full-length cDNA cloning. Three pairs of degenerate primers (P1, P2, and P3) were designed according to the conserved regions of plant LOXs sequences. P1 primers comprised LOX-S1 (5'-ATGAAGAARAATGTKTTGGAYTT-3') corresponding to the conserved amino acid region "MKKNVLDF" and LOX-A1 (5'-TTRAGGTAGAAYTCACTRTGRTG-3') corresponding to the conserved amino acid sequence region "HHSEFYLK". P2 primers comprised LOX-S2 (5'-ATYTATGTNCCRAGAGAYGA-3') encoding "IYVPRDE" and LOX-A2 (5'-TCTTCRTCHGTYCKCCAWGCAG-3') encoding "TAWRTDEE". P3 primers comprised LOX-S3 (5'-CATGGYGACAARAAAGAYGAGCC-3') encoding "HGDKKHE" and LOX-A3 (5'-ATTGARACACTRTTRGGWATTCC-3'; R = A, G; K = G, T; Y = C, T; N = A, T, C, G; H = A, C, T; W = A, T) encoding "GIPNSVSI". PCR were performed using each pair of the primers separately and the central regions of tea plant *CsLOX1 *cDNA were amplified. Full-length cDNA of *CsLOX1 *was obtained by 5' and 3' RACE approaches. For 3' RACE, two gene-specific forward primers (L3-1F: 5'-TGTCAAGGCATTCGTCAG-3' and L3-2F: 5'-CATTCGTCAGATGAGGTC-3') associated with an oligo(dT)_18 _primer were used to amplify the 3' end. The 5' RACE-PCR was carried out as recommended by the manufacturer of the commercial kit (Takara, Japan). A combination of gene-specific reverse primers (L5-1R:5'-GTCGGCATTAACTGCACTTATGAG-3' and L5-2R:5'-GCATTGAAGTCGTTAAAGTCCAA-3') and the adapter oligonucleotide provided in the kit was used to amplify the 5' end containing the 5'-UTR. To confirm that all the segments were derived from the same cDNA transcripts, two primers were designed to amplify the full-length *CsLOX1 *cDNA. The upstream primer LOX-U: 5'-TTGCTTGTCACTTTTTTTTGGTA-3' was designed based on the sequence of the 5' RACE product, while the downstream primer, LOX-D: 5'-TCAAATTGAAACACTATTAGGAA-3', was designed from the sequence of the 3' RACE product. All PCR products of interest were purified, cloned into the pGEM-T Easy Vector (Promega, Madison, WI, USA), and sequenced.

### Sequence analysis, alignment, and phylogenetic relationship

The identity search of *CsLOX1 *full-length cDNA and its deduced protein was carried out initially using BLAST, a database available at the website of the National Center for Biotechnology Information http://www.ncbi.nlm.nih.gov/BLAST/. The *CsLOX1 *sequence was then aligned with the sequences of other plant LOXs using ClustalW software http://www.ebi.ac.uk/clustalw/. The phylogenetic tree was constructed by the neighbour-joining method, and a bootstrap value was calculated from 1000 replicates using MEGA 4.0 http://www.megasoftware.net/index.html.

### Heterologous expression and purification of CsLOX1 in Yeast

The ORF of the *CsLOX1 *cDNA was amplified by PCR using the oligonucleotide primers 5'-GCGTACGTAATGTTGCACAGGGTTGTGGA-3' and 5'-GTCGCGGCCGCTCAAATTGAAACACTATTAGG-3'. These primers introduced a SnaB I and a Not I site (underlined) at the *CsLOX1 *ORF 5' and 3' ends, respectively. The PCR product was digested with the restriction enzymes SnaB I and Not I and ligated to *P. pastoris *expression vector pPIC9K (Invitrogen, Carlsbad, CA, USA), which was also digested with the same enzymes. The recombinant plasmid harbouring the yeast α-signal in frame with *CsLOX1 *coding sequences was validated by sequence analysis and named pPIC9K/*lox1*. *P. pastoris *strain GS115 was transformed with 10 μg of Sac I-linearized recombinant plasmid pPIC9K/*lox1 *using a Gene Pulser electroporator (BioRad, Hercules, CA, USA) and subsequently plated on minimal methanol agar substrate. The empty pPIC9K vector was transferred into GS115 *Pichia *host strain as control.

The recombinant colonies were first grown in 5 ml BMGY (1% yeast extract, 2% peptone, 0.1 M potassium phosphate [pH 6.0], 13.4 g/l of yeast nitrogen base without amino acids, 400 μg/l biotin, and 1% [v/v] glycerol) at 30°C until the log phase growth (OD_600 _= 2~6). Cells were harvested, washed, and suspended in BMMY (the same composition as above except that 0.5% [v/v] methanol replaced the glycerol) to a final concentration of 1 OD_600_. CsLOX1 biosynthesis was induced by adding 0.5% (v/v) methanol in BMMY for 5 d and then measured.

The culture filtrate was centrifuged at 10 000 *g *for 10 min at 4°C and the resultant supernatant was used as a crude enzyme extract preparation and later purified. Solid ammonium sulphate was added to the culture filtrate to 70% saturation. After 12 h the resulting precipitate was collected by centrifuging at 8000 *g *for 15 min, dissolved in a buffer (50 mM Tris-HCl, pH 8.0), and dialyzed overnight during which the buffer was changed three times. Insoluble material was removed by centrifuging at 10 000 *g *for 10 min. Ion-exchange chromatography was carried out using DEAE-sepharose: the dialyzed sample was put on a DEAE-sepharose column (1 × 20 cm) equilibrated with the same buffer. After the column had been washed with five column volumes of the buffer, a linear gradient of NaCl (0-0.3 M in 50 mM Tris-HCl, pH 8.0) was applied at a flow rate of 60 ml/h. Fractions with lipoxygenase activity were pooled for the determination of purity and other properties. The enzyme was confirmed by SDS-polyacrylamide gel electrophoresis (SDS-PAGE) and assayed for LOX activity.

### LOX activity

LOX activity was determined spectrophotometrically by continuously monitoring the increase in *A*_234 _resulting from the formation of conjugated diene structures during the oxidation of polyunsaturated fatty acids. One unit of LOX activity was the amount of enzyme required to produce 1 μmol of HPO and hydroxy acid per min at 25°C [49].

Protein measurement was performed as described by Bradford [50] using bovine serum albumin as a standard. Each sample had three replicates and each measurement was repeated three times. For LOX activity in the flowers, total protein was extracted from approximately 1 g of floral tissue in a pestle and mortar with sodium acetate buffer (pH 5.0) containing 1% (w/v) polyvinylpolypyrrolidone at 4°C. The homogenate was centrifuged at 12 000 *g *for 10 min at 4°C, and the supernatant was collected as the crude enzyme source and stored at -70°C for analysing LOX activity.

### Characterization of the recombinant CsLOX1

The pH-activity profiles of recombinant CsLOX1 were measured at different pH values. The following buffers were used at 50 mM: KCl-HCl (pH 2.0), glycin-HCl (pH 2.2-3.6), CH_3_COOH-CH_3_COONa (pH 3.8, 4.0, 5.0), Na_2_HPO_4_-NaH_2_PO_4 _(pH 6.0, 7.0), and Tris-HCl (pH 8.0). The reaction mixture (3 ml) consisted of 0.3 mM linoleic acid in 50 mM buffer with different pH values and 0.1 ml purified enzyme solution. The control reaction was carried out using the same pH buffer but using water instead of the enzyme. The increased *A*_234 _was monitored in the spectrophotometer for 5 min. The slope of the linear portion of the plot was used to calculate the LOX activity. For the optimal temperature analysis, the LOX activities were measured in 50 mM buffer over a temperature range of 15-50°C in increments of 5°C. Each sample consisted of 3 replicates. The maximum activity was estimated to be 100%. The kinetic parameters had been determined from a Michaelis-Menton plot in a range of substrate concentrations (linoleic acid and linolenic acid) between 75 and 600 μM.

For analysing the CsLOX1 products, the reaction mixture of enzyme activity was stopped by adding 0.1 M HCl solution, n-hexane was then added to the mixture, and the products were extracted by shaking. The 9-HPOD and 13-HPOD isomers were analysed using a Waters series system (Milford, MA, USA) with a silica ultrasphere column (Inertsil^® ^SIL-100A, 250 × 4.6 mm, 5 μm particle size). The elutant consisted of n-hexane: 2-propanol: acetic acid (100:5:0.1, v/v/v) at 1 ml/min and the effluent was monitored at 234 nm. Enantiomer composition analysis was carried out using chiral-phase HPLC on a Chiralcel OBH column (Daicel Chem. Industries, 250 × 4.6 mm, 5 μm particle size); the solvent system comprised hexane:2-propanol:acetic acid (100:5:0.1, v/v/v) and a flow rate of 1 ml/min. Standards of 9- and 13-HPOD were purchased from Larodan (Malm, Sweden).

### RNA extraction and quantitative real-time RT-PCR analysis

Total RNAs from roots, stems, leaves, seeds, flowers (petals, stamens and pistils), petals at different stages, herbivore-infested leaves, mechanically wounded leaves, and hormone-treated leaves were extracted using Trizol following the manufacturer's instructions (Invitrogen, Carlsbad, CA, USA). cDNA was prepared using 2 μg DNase-treated RNA, oligo(dT)_18 _primer, and Superscript III Polymerase (Invitrogen) to a total volume of 20 μl. The cDNA was diluted 1:10 with water, and 2 μl of the diluted cDNA was used as a template for real-time PCR experiments.

Real-time PCR reactions were perfomed in a total volume of 50 μl, 0.4 μM of each primer and 25 μl of 2 × SYBR Green PCR Master Mix (Takara, Japan) on an ABI 7500 sequence detection system (Applied Biosystems, USA). The PCR program consisted of a preliminary step of 1 min at 95°C followed by 40 cycles at 95°C for 15 s and at 60°C for 34 s. No-template controls for each primer pair were included in each run. The primers were designed based on the sequence of *CsLOX1 *ORF and 3'-UTR, the upstream primer: 5'-GCTGACTGGACAACCGATGA -3', and the downstream primer: 5'-CAACATATGCTTCTATGAAAATGC-3'. The oligonucleotides used for control expression analysis were designed on the basis of *GAPDH*, the upstream primer: 5'-ATACCACGTCATCCTCGGT-3', and the downstream primer: 5'-ACTTATGATGAAATCAAAGCTGC -3', and the product was about 100 bp. At least three different RNA isolations and cDNA syntheses were used as replicates for the qPCR. The *LOX/GAPDH *ratios for all samples were related to the ratio for untreated plants, which was set to 1.

### Collection and analysis of volatiles

Before collecting the volatiles from each infested plant, the insects were carefully removed. The intact and insect challenged plants were separately placed in a 25 l glass vessel with an inlet and an outlet. Dried and purified air was guided through the inlet at 250 ml/min for 48 h. A Tenax TA column with 150 mg absorbent (Supelco, Inc., Bellefonte, PA, USA) activated under 275°C with nitrogen for 4 h was attached to the outlet. Volatiles were desorbed by eluting the Tenax traps with 1 ml freshly distilled hexane. Then 20 μl of a 100 μg/ml decanoic acid ethyl ester were added as an internal standard. The resulting extract was concentrated to about 20 μl under a stream of nitrogen, 2 μl of which were directly analyzed by GC-MS on a Trace 2000 gas chromatograph fitted with an apolar column DB-5MS (30 m × 0.25 mm, film thickness 0.25 um, Agilent Technologies, Santa Clara, California, USA) coupled with a Trace DSQ mass selective detector (MS). The oven temperature was programmed as follows: 50°C for 5 min, raised to 180°C at 2°C/min, and finally raised to 230°C at 10°C/min and held for 10 min. The injection was splitless and the injector temperature was 230°C. The mass spectra were obtained under electron ionization impact at 70 eV and data acquisition was done under a full scan MS mode. Identifications were confirmed with retention time and peak enhancement on co-injection with authentic commercial samples. Quantifications of volatiles were confirmed by comparison of their peak area with that of the internal standard.

## Authors' contributions

SL isolated the *CsLOX1 *gene, transformed the gene into yeast and characterized the recombinant protein, carried out quantitative RT-PCR, analysed the *CsLOX1 *expression pattern during flower development and the response of leaves to attack by different phloem-feeders, and wrote the manuscript. BH collected and analysed the volatiles from intact and insect-damaged tea plants. Both SL and BH coordinated the project and edited the final manuscript, which was approved by both the authors.

## Supplementary Material

Additional file 1 **Nucleotide sequence of *CsLOX1 *cDNA with translation of the coding region containing nucleotide sequences, amino acid sequences, typical functional domains of LOXs, and amino acids for degenerate primers design**. The substrate-binding domains, the oxygen-binding domains, and the conserved C-terminal amino acid sequences are shown against a grey background; the highly conserved histidine residue is in bold; and the amino acids for degenerate primers design are underlined. Click here for file
