# Supplementary material for: Differential expression pattern of an acidic 9/13-lipoxygenase in flower opening and senescence and in leaf response to phloem feeders in the tea plant
Source: BMC Plant Biol. 2010 Oct 25;10:228. doi: 10.1186/1471-2229-10-228 (PMC3095316; doi:10.1186/1471-2229-10-228)
Supplement: Additional file 1 — Nucleotide sequence of CsLOX1 cDNA with translation of the coding region containing nucleotide sequences, amino acid sequences, typical functional domains of LOXs, and amino acids for degenerate primers design. The substrate-binding domains, the oxygen-binding domains, and the conserved C-terminal amino acid sequences are shown against a grey background; the highly conserved histidine residue is in bold; and the amino acids for degenerate primers design are underlined. [file 1471-2229-10-228-S1.DOC]

1 GAAAATTCATTCATTTGTACCAACAAGAA

30 CAACAATATATAGCTCTTTGTTTTGCAAGTTTGCGAAAATATTCTTTGCTTGTC

84 ACTTTTTTTTGGTATTTTCTTTTTTGTTTTCTCGAGAAAACAAGAGAGATAGAG

138 ATGTTGCACAGGGTTGTGGAGGGGATCAAAGGCAACGATGGGAATGATAAGAAG

1 M L H R V V E G I K G N D G N D K K

192 ATCAAAGGGACTGTTGTGTTGATGAAGAAGAATGTTTTGGATTTCAATGACTTC

19 I K G T V V L M K K N V L D F N D F

246 AATGCTTCCATTCTTGATCGGGTTCATGAGTTGCTTGGACAAAAAGTCTCTTTG

37 N A S I L D R V H E L L G Q K V S L

300 CAACTCATAAGTGCAGTTAATGCCGACCTTACTGTGAAAGGGCTGAAAGGGAAA

55 Q L I S A V N A D L T V K G L K G K

354 CTTGGAAAGCCTGCATATTTGGAAGACTGGATTACTACAATCACCCCATTAACT

73 L G K P A Y L E D W I T T I T P L T

408 GCAGGCGATTCAGCATACGATGTCACATTTGATTGGGACGAGGAGATAGGAGTT

91 A G D S A Y D V T F D W D E E I G V

462 CCAGGGGCATTCATAATAAGAAACTTTCACCATAGTGAATTCTATCTTAAGTCT

109 P G A F I I R N F H H S E F Y L K S

516 CTCACACTTGATCACGTTCCTGGACACGGTCGCGTTCACTTTGTGTGCAACTCT

127 L T L D H V P G H G R V H F V C N S

570 TGGGTTTACCCAGCCAAAAATTACAAAACAGATCGTGTTTTCTTCAGCAATCAA

145 W V Y P A K N Y K T D R V F F S N Q

624 ACGTATCTTCTAAGTGAAACTCCGGCACCGCTCATTGAGTACAGAAAACAAGAA

163 T Y L L S E T P A P L I E Y R K Q E

678 CTAGTGAACTTGAGAGGAGATGGAAAAGGAAAGCTTGAGGAATGGGACAGGGTT

181 L V N L R G D G K G K L E E W D R V

732 TATGACTATGCTTACTACAATGACTTGGGAGATCCCGATAAGGGCTCCAAATAT

199 Y D Y A Y Y N D L G D P D K G S K Y

786 GCCCGCCCAATTCTTGGTGGATCGACAGAGTACCCTTATCCCCGTAGGGGAAGA

217 A R P I L G G S T E Y P Y P R R G R

840 ACAGGCCGCCCACCAACCAAGACAGATCCAGAATCGGAGAGTAGGTTGGCGCTT

235 T G R P P T K T D P E S E S R L A L

894 CTAATGAGCTTCAACATTTATGTTCCAAGAGATGAACGGTTTGGTCACTTGAAG

253 L M S F N I Y V P R D E R F G H L K

948 ATGTCAGATTTCTTAGCGTATGCATTGAAATCCGTTGTTCAATTCCTTGTTCCA

271 M S D F L A Y A L K S V V Q F L V P

1002 GAGCTTGGGGCTTTATGTGATAAAACACCAAATGAGTTTGATTCTTTCCAAGAT

289 E L G A L C D K T P N E F D S F Q D

1056 ATACTCAAAATCTATGAAGGAGGAATCAAGCTGCCTGAGGGGCCTTTACTTGAC

307 I L K I Y E G G I K L P E G P L L D

1110 AAGATTAAAGAAAATATTCCCTTGGAAATGCTCAAGGAACTCGTGAGGACCGAT

325 K I K E N I P L E M L K E L V R T D

1164 GGCGAGGGATACCTCAAATTCCCAATGCCACAAGTGATAAAAGAGGACAAGACT

343 G E G Y L K F P M P Q V I K E D K T

1218 GCATGGCGGACAGACGAAGAATTTGCAAGAGAAATGTTGGCTGGAGTAGACCCT

361 A W R T D E E F A R E M L A G V D P

1272 GTCATCATTAGTCGTCTCCAAGAGTTCCCTCCAAGAAGCACTCTAGATCCTAAA

379 V I I S R L Q E F P P R S T L D P K

1326 CTCTATGGCAATCAAAACAGTTCAATAACTGAAGATCACATAAAGAACAATCTA

397 L Y G N Q N S S I T E D H I K N N L

1380 GATGGGTTCACAATAGAAGAGGCAATCAAAAACAACCGGCTCTTCATATTAGAT

415 D G F T I E E A I K N N R L F I L D

1434 CACCACGATGCTTTAATGCCATATGTAAGGCGGATTAATGCAACATCCACAAAG

433 H H D A L M P Y V R R I N A T S T K

1488 ATTTACGCCACAAGAACTCTCCTCTTCCTGCAAAAAGACGGGACTTTGAAGCCT

451 I Y A T R T L L F L Q K D G T L K P

1542 CTGGCAATCGAATTAAGCTTGCCACACCCAAATGGAGATCAGTTTGGTGCCATA

469 L A I E L S L P H P N G D Q F G A I

1596 AGCAAAGTGTACACCCCATCTGAGCAGGGTGTTGAAGGCTCCGTTTGGCAATTG

487 S K V Y T P S E Q G V E G S V W Q L

1690 GCTAAAGCATATGTTGCAGTCAATGATTCCGGCTACCATCAGCTCATCAGCCAT

505 A K A Y V A V N D S G Y **H** Q L I S **H**

1704 TGGTTGAATACTCATGCGGCAATTGAGCCATTTGTGACCGCAACAAATAGACAG

523 W L N T  **H**  A A I E P F V T A T N R Q

1758 CTGAGTGTGCTTCACCCGATACACAAGCTTTTGCATCCTCACTTCCGTGATACA

541 L S V L **H** P I H K L L H P **H** F R D T

1812 ATGAATATAAATGCTTTTGCTAGACAAATCTTGATTAATGCCGATGGAATTCTT

559 M N I N A F A R Q I L I N A D G I L

1866 GAGAAAACAGTTTTTCCAGGAAAGTATGCCATGGAAATGTCGGCTGTAGTCTAC

577 E K T V F P G K Y A M E M S A V V Y

1920 AAGAACTGGGTTTTTCCCGAGCAAGCACTTCCTGCCGATCTTATCAAGAGAGGA

595 K N W V F P E Q A L P A D L I K R G

1974 GTGGCGGTGAAGGACGACAACGCTCCACATGGCATCCGATTATTGATTCAAGAC

613 V A V K D D N A P H G I R L L I Q D

2028 TGCCCTTATGCAGTGGATGGACTTAAAATTTGGTCAGCAATCGAAACTTGGGTT

631 C P Y A V D G L K I W S A I E T W V

2082 CAGGAGTACTGTAATTTTTACTACAAGAATGACGAAATGGTTAAGGAAGACTTA

649 Q E Y C N F Y Y K N D E M V K E D L

2136 GAACTCCAATCTTGGTGGAAGGAATTGAGAGAGGAAGGGCATGGCGACAAGAAA

667 E L Q S W W K E L R E E G H G D K K

2190 CACGAGCCATGGTGGCCTAAAATGCAGACTCGAAGAGAGCTAATTGACTCTTGC

685 H E P W W P K M Q T R R E L I D S C

2244 ACGATTGTTATTTGGGTGGCTTCTGCCCTCCACGCTGCTGTCAATTTTGGACAG

703 T I V I W V A S A L H A A V N F G Q

2298 TACCCTTATGCAGGGTACCTCCCAAACCGCCCAACTCTAAGTCGTCGGTTCATG

721 Y P Y A G Y L P N R P T L S R R F M

2352 CCTGAACCTGGTACTCCTGAGTACGAAGAGTTCAAGTCGAGCCCTGACAAGGCT

739 P E P G T P E Y E E F K S S P D K A

2406 TTCTTGAAAACAATCACAGCCCAGTTGCAAACACTTCTTGGGGTTTCTTTGATT

757 F L K T I T A Q L Q T L L G V S L I

2460 GAGATTTTGTCAAGGCATTCGTCAGATGAGGTCTATCTTGGGCAAAGAGACAGT

775 E I L S R H S S D E V Y L G Q R D S

2514 GCTGACTGGACAACCGATGACGAACCATTGGAGGCATTTGGAAGATTTGGGAAA

793 A D W T T D D E P L E A F G R F G K

2568 AAATTGGGAGAGATTGAAGAAATGATCATTGAAATGAACAATGATGAGAATTTG

811 K L G E I E E M I I E M N N D E N L

2662 AGGAACAGGGTTGGGCCTGTTAAGGTTCCATATACTCTGTTGTTTCCAACAAGT

829 R N R V G P V K V P Y T L L F P T S

2676 GAAGGTGGACTCACTGGAAAGGGAATTCCTAATAGTGTTTCAATTTGAATATTT

847 E G G L T G K G I P N S V S I *

2730 TCTTTCTTTTGTATTCTGATGTTGCATTTTCATAGAAGCATATGTTGATTTATA

2784 AAAAAAAAAAAAA
